# Análise Crítica e Limitações do Diagnóstico de Insuficiência Cardíaca com Fração de Ejeção Preservada (ICFEp)

**DOI:** 10.36660/abc.20210052

**Published:** 2022-07-07

**Authors:** Viviane Tiemi Hotta, Daniela do Carmo Rassi, José Luiz Barros Pena, Marcelo Luiz Campos Vieira, Ana Clara Tude Rodrigues, Juliano Novaes Cardoso, Felix Jose Alvarez Ramires, Luciano Nastari, Charles Mady, Fábio Fernandes

**Affiliations:** 1 Hospital das Clínicas Faculdade de Medicina Universidade de São Paulo São Paulo SP Brasil Instituto do Coração, Hospital das Clínicas da Faculdade de Medicina da Universidade de São Paulo (InCor/HCFMUSP), São Paulo, SP – Brasil; 2 Fleury Medicina e Saúde São Paulo SP Brasil Fleury Medicina e Saúde, Ecocardiografia, São Paulo, SP – Brasil; 3 Faculdade de Medicina Universidade Federal de Goiás Goiânia GO Brasil Faculdade de Medicina da Universidade Federal de Goiás, Goiânia, GO – Brasil; 4 Faculdade de Ciências Médicas de Minas Gerais Belo Horizonte MG Brasil Faculdade de Ciências Médicas de Minas Gerais, Belo Horizonte, MG – Brasil; 5 Hospital Felício Rocho Belo Horizonte MG Brasil Hospital Felício Rocho, Belo Horizonte, MG – Brasil; 6 Hospital das Clínicas Faculdade de Medicina Universidade de São Paulo São Paulo SP Brasil Hospital das Clínicas da Faculdade de Medicina da Universidade de São Paulo (HCFMUSP), São Paulo, SP – Brasil; 7 Hospital Israelita Albert Einstein São Paulo SP Brasil Hospital Israelita Albert Einstein, São Paulo, SP – Brasil

**Keywords:** Insuficiência Cardíaca/fisiopatologia, Diagnóstico por Imagem, Ecocardiografia/métodos, Peptídeos Natriuréticos

## Abstract

Com o aumento da expectativa de vida da população e a maior frequência de fatores de risco como obesidade, hipertensão arterial e diabetes, espera-se um aumento na prevalência de insuficiência cardíaca com fração de ejeção preservada (ICFEp). Entretanto, no momento, o diagnóstico e o tratamento de pacientes com ICFEp permanecem desafiadores. O diagnóstico sindrômico de ICFEp inclui diversas etiologias e doenças com tratamentos específicos, mas que apresentam pontos em comum em relação à apresentação clínica e à avaliação laboratorial no que diz respeito aos biomarcadores como BNP e NT-ProBNP, à avaliação ecocardiográfica do remodelamento cardíaco e às pressões de enchimento diastólico ventricular esquerdo. Extensos ensaios clínicos randomizados envolvendo a terapia nesta síndrome falharam na demonstração de benefícios para o paciente, fazendo-se necessária uma reflexão acerca do diagnóstico, dos mecanismos de morbidade, da taxa de mortalidade e da reversibilidade. Na revisão, serão abordados os conceitos atuais, as controvérsias e, especialmente, os desafios no diagnóstico da ICFEp através de uma análise crítica do escore da *European Heart Failure Association.*

## Introdução

Estima-se que, na maioria da população acima de 60 anos de idade, cerca de 5% dos pacientes apresentam diagnóstico de insuficiência cardíaca com fração de ejeção preservada (ICFEp), sendo que a prevalência varia entre 3,8 a 7,4% entre os estudos realizados, considerando-se as diferentes metodologias no diagnóstico.^1^ Com o aumento da expectativa de vida da população e a maior frequência de fatores de risco como obesidade, hipertensão arterial e diabetes, espera-se um aumento na prevalência de ICFEp.^2 - 4^

Entretanto, até o momento, o diagnóstico e o tratamento de pacientes com ICFEp permanecem desafiadores. O diagnóstico sindrômico de ICFEp inclui diversas etiologias e doenças com tratamentos específicos, mas que apresentam pontos em comum em relação à apresentação clínica, à avaliação laboratorial no que diz respeito aos biomarcadores como BNP e NT-ProBNP e à avaliação ecocardiográfica do remodelamento cardíaco e das pressões de enchimento diastólico ventricular esquerdo.^1^ Ao contrário da IC-FEr, nenhum tratamento mostrou de forma convincente a redução da morbidade ou mortalidade na ICFEp, fazendo-se necessária uma reflexão acerca do diagnóstico, dos mecanismos de morbidade, da mortalidade e da reversibilidade nesta síndrome.^5^

Na revisão, serão abordados os conceitos atuais, controvérsias e, especialmente, os desafios no diagnóstico da ICFEp, analisando criticamente o escore da *European Heart Failure Association* .^1^

### Escore da *European Heart Failure Association* para o diagnóstico da ICFEp

Em 2019, foi publicado pela *Heart Failure Association* (HFA), da *European Society of Cardiology* (ESC), um novo posicionamento para o diagnóstico de ICFEp, que inclui o papel das comorbidades clínicas e um sistema fundamentado em escore com valores atualizados dos critérios ecocardiográficos e a dosagem de biomarcadores, além do papel dos testes feitos sob esforço ( Tabela 1 ).^6 - 8^

**Tabela 1:** Algoritmo *Heart Failure Association* (HFA) para o diagnóstico de ICFEp, da *European Society of Cardiology* (ESC)

P	Avaliação inicial	Comorbidades/ Fatores de risco
	Passo 1 (P): avaliação pré-teste	Sinais e ou sintomas de IC
		Eletrocardiograma
		Avaliação ecocardiográfica convencional
		Dosagem de peptídeos natriuréticos
		Teste ergométrico, teste de caminhada de 6 minutos ou cardiopulmonar, em casos selecionados
**E**	Avaliação diagnóstica	Avaliação ecocardiográfica dedicada
	Passo 2 (E): escore ecocardiográfico e peptídeos natriuréticos	
**F1**	Avaliação avançada	Teste diagnóstico sob estresse: ecocardiografia sob estresse físico
	Passo 3 (F1): teste funcional em caso de incertezas	Medidas hemodinâmicas invasivas
**F2**	Avaliação etiológica final	Ressonância magnética cardíaca
	Passo 4 (F2): Etiologia final	Biópsia cardíaca e extra cardíaca
		Cintilografia/CT/ PET CT
		Testes genéticos
		Testes laboratorias específicos

*IC: insuficiência cardíaca; CT: tomografia computadorizada; PET CT: Tomografia por Emissão de Pósitrons. Adaptado de Pieske B et al.^1^*

A avaliação inicial deve levar em consideração a anamnese, abordando os fatores de risco, as comorbidades, a presença de sintomas e os sinais relacionados ao exame físico de insuficiência cardíaca que sugiram o diagnóstico de ICFEp, conforme o diagrama a seguir ( Tabela 1 ). Nessa fase inicial, devem ser realizados exames de sangue, incluindo peptídeos natriuréticos (PN), eletrocardiograma, teste ergométrico, teste de caminhada de seis minutos e/ou teste cardiopulmonar, além de avaliação ecocardiográfica.^1^

O eletrocardiograma (ECG) pode evidenciar sinais de hipertrofia ventricular esquerda (índice de Sokolow-Lyon; índice ≥3,5 mV) e/ou sobrecarga atrial esquerda, mas sua principal indicação é detectar a presença de fibrilação atrial (FA), altamente preditiva de ICFEp subjacente.^9 , 10^

O racional para o emprego do escore baseia-se no fato de que nenhum critério não invasivo é suficiente de maneira isolada para o diagnóstico de ICFEp. Por isso, recomenda-se a avaliação integrada das informações clínicas, das medidas dos níveis séricos de PN e da estrutura e função cardíaca pela ecocardiografia.^10^ É importante lembrar que os valores de corte podem variar de acordo com a idade, gênero, peso corporal, função renal e presença de FA. Assim, são recomendados critérios menores e maiores de acordo com o grau de alteração na presença dos fatores modificadores acima descritos.^1^

Valores de PN em pacientes em ritmo de FA podem ser até três vezes maiores do que em pacientes em ritmo sinusal. Por isso, os valores de corte são diferenciados para as duas populações de pacientes.^11 , 12^ Até o momento, valores de corte definitivos para o diagnóstico de ICFEp em pacientes em ritmo sinusal ou FA não foram bem estabelecidos.^1^ Os valores sugeridos para o diagnóstico de ICFEp são descritos na Figura 2 .

**Figura 2:**
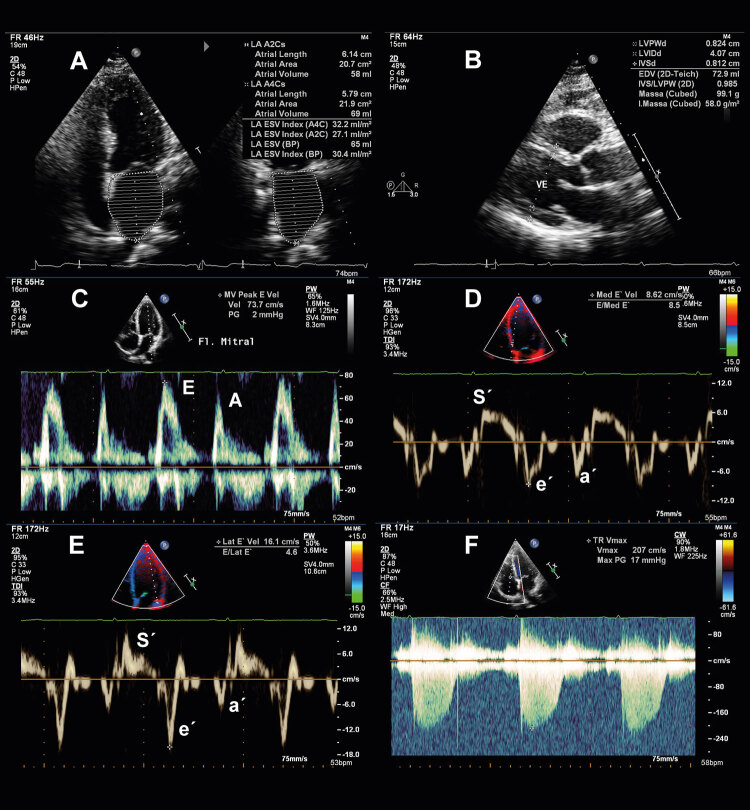
Critérios ecocardiográficos morfológicos (A e B) e funcionais (C a F) para aplicação do algoritmo diagnóstico para pacientes com suspeita de ICFEp. Os critérios morfológicos incluem a medida do volume indexado do átrio esquerdo (A), e o cálculo do índice de massa miocárdica e a espessura relativa de parede (B). Os critérios funcionais incluem a relação E/e’, calculada a partir da medida de onda E ao Doppler mitral (C) (v = 73,7 cm/s) e ondas e’ ao Doppler tecidual septal (D) (v = 8,6 cm/s) e lateral (E) (v = 16,1 cm/s), além da velocidade de pico do jato de regurgitação tricúspide (v = 2,07 cm/s) para estimar a medida da pressão sistólica de artéria pulmonar (F). v: velocidade.

### Avaliação ecocardiográfica

A ecocardiografia é o método de imagem cardíaca de escolha na avaliação do paciente com sinais e sintomas de IC. O ecocardiograma permite a avaliação anatômica funcional pelas medidas dos diâmetros e volumes das cavidades cardíacas, estimativa da massa ventricular esquerda e análise da função sistólica pela fração de ejeção, além da função longitudinal global e segmentar miocárdica. É o método não invasivo de escolha para a análise da função diastólica e das pressões de enchimento ventricular esquerdo e da artéria pulmonar.^1^

### Critérios morfológicos

#### Medidas do volume atrial esquerdo indexado (VolAEi)

O VolAEi está relacionado às pressões de enchimento do VE e a outros índices de função diastólica, sendo a medida mais acurada do remodelamento crônico do AE quando é comparada ao diâmetro e área do AE.^13 , 14^

Em pacientes sem FA ou doença valvar cardíaca, o VolAEi >34 ml/m^2^ é preditor independente de morte, IC, FA e acidente vascular encefálico isquêmico.^15 , 16^ Em pacientes com ICFEp e FA permanente, o VolAEi foi 35% maior do que nos pacientes com ICFEp em ritmo sinusal.^11^ Pacientes com FA permanente podem apresentar maiores VolAEi, mesmo na ausência de disfunção diastólica. Assim, recomenda-se valores de corte distintos de VolAEi para o diagnóstico de ICFEp de pacientes em ritmo sinusal e FA ( Figuras 1 e 2 ).^15 , 16^

**Figura 1:**
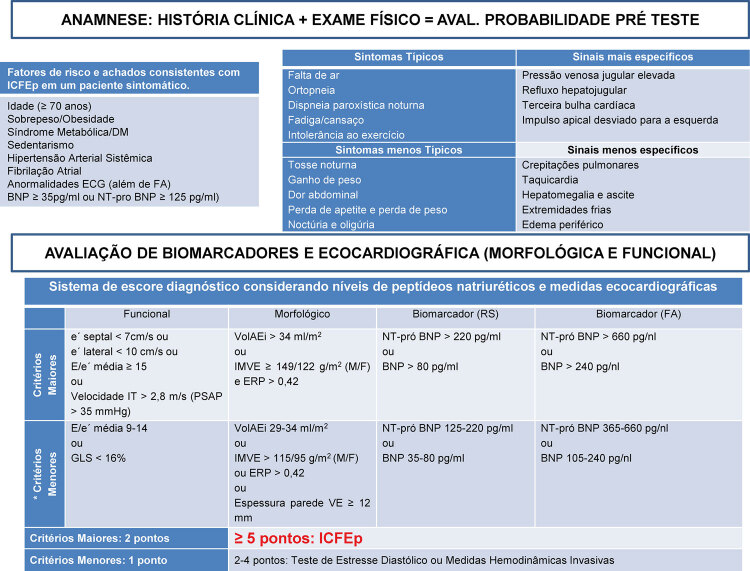
Fluxograma para avaliação clínica, integrando fatores de risco, exame físico, avaliação de biomarcadores e análise ecocardiográfica. DM: diabetes mellitus; ECG: eletrocardiograma; FA: fibrilação atrial; IT: insuficiência tricúspide; PSAP: pressão sistólica em artéria pulmonar; VolAEi: volume do átrio esquerdo indexado; IMVE: índice de massa do ventrículo esquerdo; ERP: espessura relativa da parede ventricular; BNP: peptídeo natriurético atrial; GLS: strain global longitudinal; ICFEp: insuficiência cardíaca com fração de ejeção preservada.* Critério menor não deve ser contabilizado dentro do mesmo domínio.

#### Medidas da espessura miocárdica e estimativa da massa ventricular esquerda

No escore da HFA, a espessura ventricular esquerda ao final da diástole das paredes septal e posterior foi considerada um critério morfológico para o diagnóstico de ICFEp.^1^ Preferencialmente, tais medidas devem ser obtidas

no modo 2D, ou no modo M guiado pelo 2D, de acordo com a fórmula recomendada pela Sociedade Americana de Ecocardiografia.^17 , 18^

O índice de IMVE é definido como a massa ventricular esquerda indexada pela superfície corpórea. A hipertrofia é o aumento do IMVE, de acordo com os seguintes valores de referência: ≥95 g/m^2^ em mulheres e ≥115 g/m^2^ em homens.^17 , 18^ É importante considerar o cálculo da espessura relativa de parede (ERP).^17 , 18^ A análise da IMVE e da ERP permite a categorização da hipertrofia em concêntrica (aumento do IMVE e ERP >0,42), excêntrica (aumento do IMVE e ERP <0,42) e remodelamento concêntrico (IMVE normal e ERP >0,42).^17 , 18^

Em pacientes com ICFEp, é possível observar os padrões de remodelamento ou hipertrofia concêntrica. No entanto, a ausência de hipertrofia ventricular esquerda não exclui o diagnóstico de ICFEp.^1^ Dessa forma, são considerados os critérios descritos na Figuras 1 e 2 .

**Figure 1:**
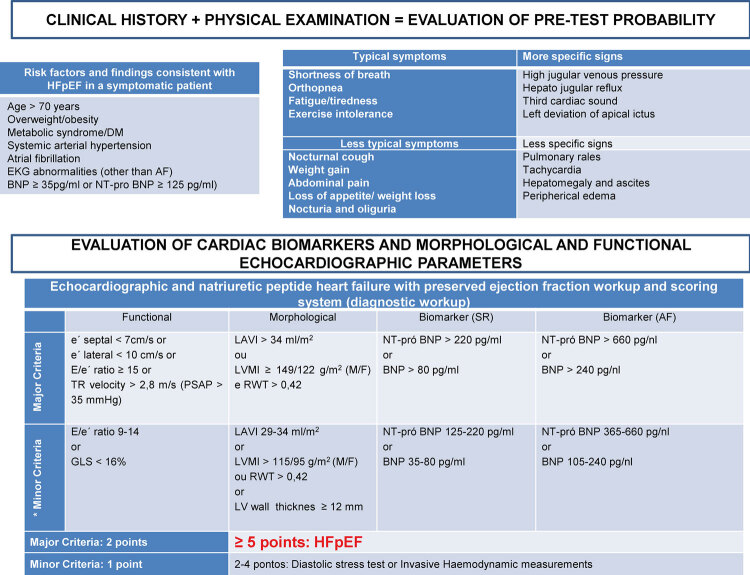
Clinical evaluation flowchart integrating risk factors, physical examination, evaluation of biomarkers and echocardiographic analysis. AF: atrial fibrillation;DM: diabetes mellitur; TR: tricuspid regurgitation; LAVI: left atrial volume index; LVMI: left ventricular mass index; RWT: left ventricular relative wall thickness; BNP: B-type natriuretic peptide; GLS: global longitudinal strain; PASP: pulmonary artery systolic pressure; HFpEF: heart failure with preserved ejection fraction. * Minor criterion should not be counted within the same domain.

**Figure 2:**
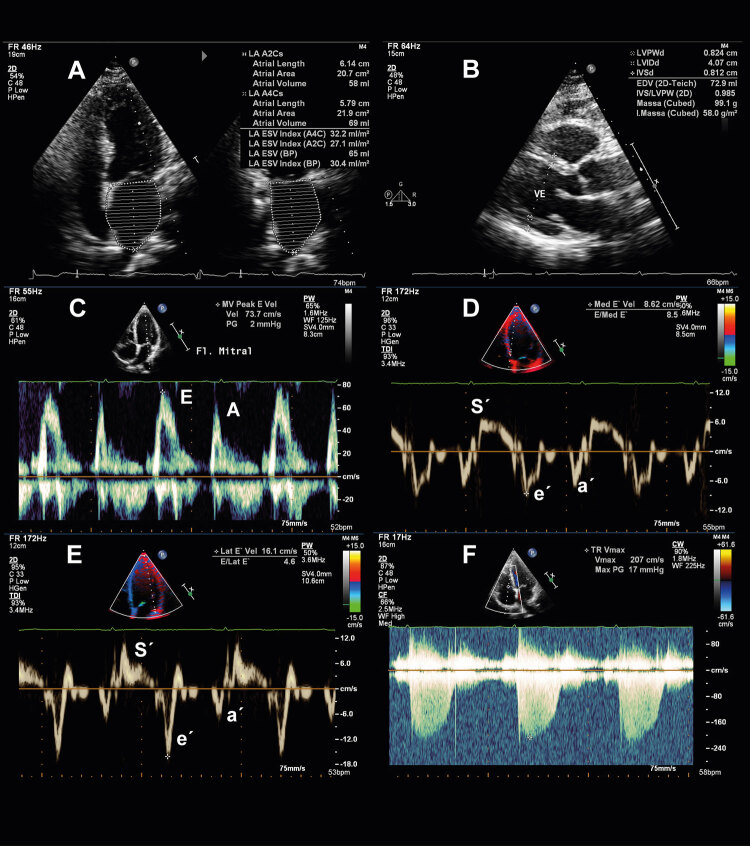
Morphological (A and B) and functional (C to F) echocardiographic criteria for the diagnostic algorithm application in patients with suspected HFpEF. The morphological criteria included the measurement of the left atrial volume index (A) and the calculation of the myocardial mass index and relative wall thickness (B). The functional criteria include the E/e´ ratio calculated from the measurement of the E wave on mitral Doppler (C) (v = 73.7 cm/s) and e´ wave septal (D) (v = 8.6 cm/s) and lateral (E) (v = 16.1 cm/s) velocities on tissue Doppler, in addition to the tricuspid regurgitation jet peak velocity (v = 2.07 cm/s) to measure the pulmonary artery systolic pressure (F). v: velocity.

## Critérios funcionais

### Medidas do *Strain* Sistólico Longitudinal Global (GLS) do VE

A medida da deformação miocárdica ( *strain* ) longitudinal global ventricular esquerda (GLS) pela técnica de *speckle tracking* independe do ângulo de insonação do ultrassom confere vantagem em relação à medida do *strain* obtida pelo *Doppler* e é considerada a técnica de eleição.^19^

É importante relatar que equipamentos de diferentes marcas podem apresentar variação entre os valores de GLS aferidos em um mesmo paciente. Um valor absoluto de GLS <16% pode ser considerado anormal e, ao mesmo tempo, um critério menor para o diagnóstico de ICFEp (ver Figura 2 ).^1^ Valores reduzidos de GLS são preditores de hospitalização por IC, morte cardiovascular ou parada cardiorrespiratória, apresentando boa correlação com a rigidez do VE e com biomarcadores.^19 - 20^

### Medidas ao *Doppler* convencional

Ao *Doppler* convencional, são utilizadas as medidas da onda E ao *Doppler* pulsátil da valva mitral para o cálculo da relação E/e’ e a velocidade de pico do jato de insuficiência tricúspide (IT) ao *Doppler* contínuo. Tais medidas são importantes nas estimativas de elevação das pressões de enchimento e, consequentemente, para o diagnóstico de ICFEp.^1 , 19 , 20^

Medidas elevadas de pressão sistólica em artéria pulmonar (PSAP) e redução da função ventricular direita são importantes preditores de mortalidade em pacientes com ICFEp. Valores da velocidade de pico do jato de insuficiência tricúspide > 2,8 m/s são marcadores indiretos de disfunção diastólica e estão associados ao diagnóstico de ICFEp.^21 - 24^

### Medidas ao *Doppler* tecidual

As medidas das velocidades de pico diastólicas precoces (ondas e’) nas paredes septal e lateral ao *Doppler* tecidual pulsátil são um parâmetro fundamental em pacientes com ICFEp.^1 , 25^ Todas as medidas devem representar a média de três ou mais ciclos cardíacos consecutivos, e, preferencialmente, devem ser realizadas as medidas das velocidades das ondas e’ septal e lateral, em particular para o cálculo da relação E/e’.^25^

O maior determinante da velocidade diastólica precoce da movimentação do anel mitral é o relaxamento do VE. A onda e’ reflete o relaxamento do VE e está influenciada pela pré-carga.^26 , 27^ A velocidade da onda e” diminui com a idade e, por isso, são recomendados valores de referência de acordo com a idade para o cálculo do escore para o diagnóstico de ICFEp ( Figuras 1 e 2 ).^28^

A relação E/e’ média das paredes septal e lateral reflete a pressão capilar pulmonar média e correlaciona-se com a rigidez ventricular esquerda e a presença de fibrose, além de ser menos dependente da idade e envelhecimento do que a onda e’.^1 , 25 , 29 , 30^ A medida também apresenta valor diagnóstico durante o esforço físico, sendo pouco influenciada por alterações volumétricas, mas influenciada pela gravidade da hipertrofia ventricular esquerda.^1 , 31 - 33^

### Avaliação diagnóstica pelo escore ecocardiográfico e peptídeos natriuréticos

O escore apresenta domínios funcionais, morfológicos e relacionados aos biomarcadores, e cada critério maior atribui 2 pontos e cada critério menor 1 ponto ( Figura 2 ). É importante lembrar que nem todos os parâmetros de cada domínio podem ser analisáveis. Um escore total ≥5 pontos é considerado diagnóstico para ICFEP enquanto escores ≤1 ponto indicam o diagnóstico muito improvável e torna mandatória a investigação de diagnósticos diferenciais.^1^ Pacientes com pontuação intermediária necessitam de avaliação adicional complementar (passo 3), conforme a seguir. Na prática e de maneira estruturada, os passos 1 e 2 podem ser resumidos no fluxograma da Figura 2 .

**Figura 3:**
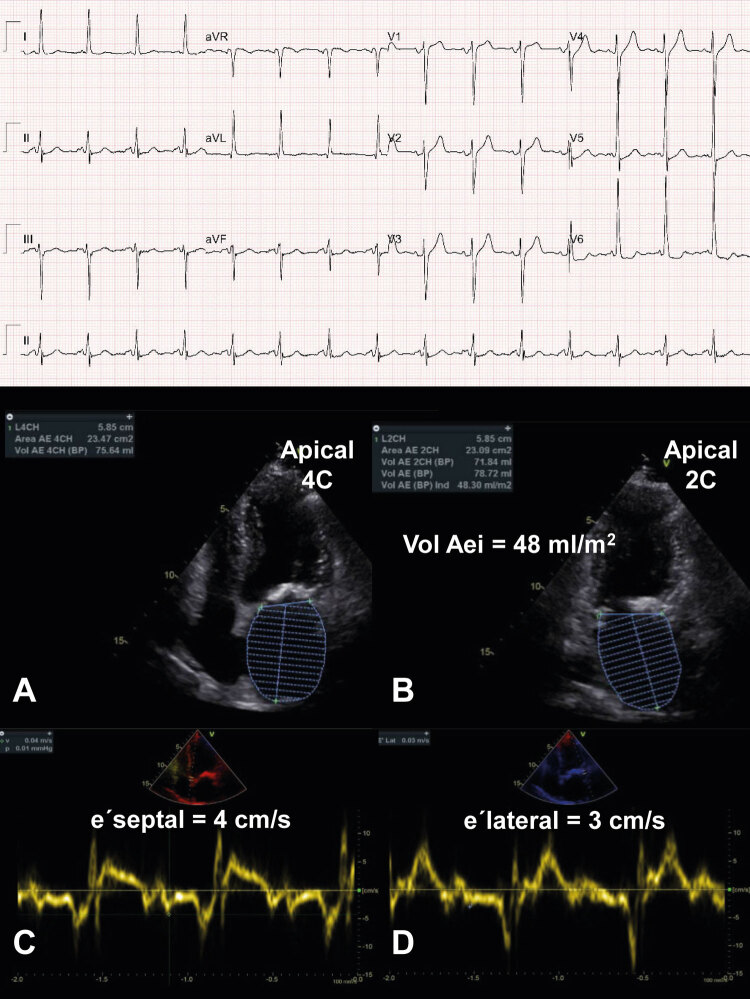
Exemplo ilustrativo de aplicação do escore diagnóstico em indivíduo com suspeita de ICFEp. Paciente do sexo feminino, 64 anos, com antecedentes de síndrome metabólica (obesidade grau III – IMC: 35,6, HAS e DM) e queixa de dispneia aos mínimos esforços (CF III NYHA). Ao ECG (acima), observa-se sinais de hipertrofia ventricular esquerda pelos critérios de Sokolow-Lyon. Apresenta ao ETT espessura do septo interventricular e parede posterior de 12 mm e IMVE: 105 g/m2 (1 ponto). Volume indexado do átrio esquerdo estimado ao corte apical 4C (acima à esquerda) e apical 2C (acima à direita) em 48 ml/m2 (2 pontos). Doppler tecidual evidencia onda e’ septal = 4 cm/s (abaixo à esquerda) e onda e’ lateral = 3 cm/s (abaixo à direita) (2 pontos). Assim, aplicando-se o escore para o diagnóstico de ICFEp, a paciente apresenta 5 pontos e, portanto, diagnóstico de ICFEp. VolAEi: volume do átrio esquerdo indexado.

Nas Figuras 3 e 4 , seguem exemplos ilustrativos da aplicação do escore em casos reais.

**Figura 4:**
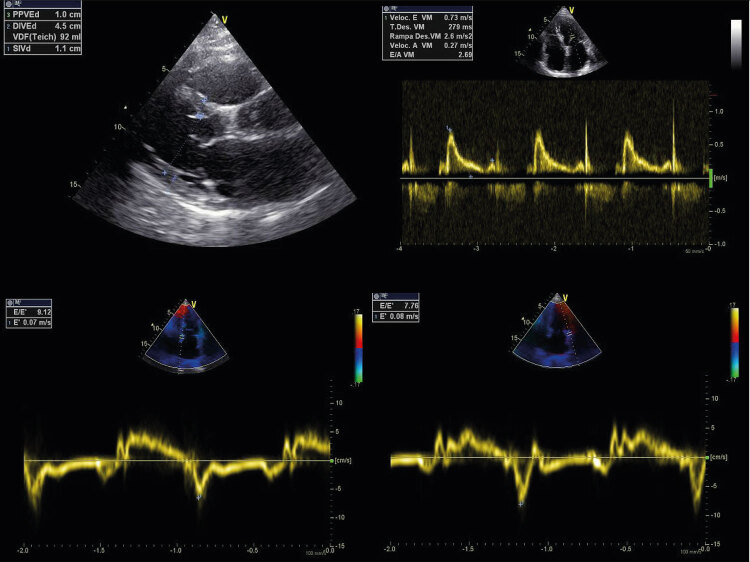
Paciente de 78 anos com obesidade, hipertensão arterial sistêmica, diabetes melito tipo 2 e fibrilação atrial paroxística em CF II NYHA. Ao ETT, apresenta AE = 50 mm, volume indexado de AE = 38 ml/m2 (2 pontos), índice de massa VE: 89 g/m2 e espessura relativa de parede = 0,47 e PSAP não analisável, relação E/e’ = 8,8 e BNP = 367 pg/ml (2 pontos). Aplicando-se o escore para o diagnóstico de ICFEp, paciente apresenta 4 pontos e, portanto, diagnóstico inconclusivo de ICFEp.

No caso real da Figura 3 , apesar de a paciente preencher critério menor morfológico de espessura relativa de parede >0,42, como já recebeu pontuação dentro do domínio morfológico por critério maior (2 pontos) pela dilatação do volume indexado, é importante salientar que o critério menor não é contabilizado dentro do mesmo domínio. A situação também é ilustrativa por evidenciar a limitação das medidas sugeridas em casos reais. Na paciente, não foi possível aferir a pressão sistólica de artéria pulmonar por conta da ausência de refluxo tricúspide, situação ocasional na prática diária.

Além disso, a paciente apresentava limitação para realizar teste sob esforço por conta de obesidade e alterações degenerativas articulares e não prosseguiu com a investigação etiológica sugerida pelo protocolo da HFA.

É importante considerar que, em pacientes com diagnóstico de estenose mitral, a onda E pode não refletir a função diastólica. Em pacientes com insuficiência tricúspide importante, a velocidade do jato de insuficiência tricúspide pode estar reduzida devido à equalização entre as pressões de VD e AD, subestimando a estimativa da PSAP.^25^

## Passo 3 (F1): avaliação avançada – teste funcional em caso de incertezas

Em pacientes com pontuação intermediária no escore diagnóstico, é indicada uma avaliação complementar com ecocardiografia e sob esforço físico, pois muitos pacientes apresentam apenas sintomas relacionados aos esforços. Dessa forma, sintomas compatíveis com ICFEp podem ser confirmados a partir de anormalidades hemodinâmicas como reduções do débito cardíaco e do volume sistólico e a elevação das pressões de enchimento do VE em repouso ou durante o esforço físico.^1 , 34^

O ecocardiograma sob esforço pode revelar disfunção sistólica e diastólica durante o exercício. Os parâmetros mais empregados para essa análise na suspeita de ICFEp são a relação E/e’ e a velocidade de pico do jato de insuficiência tricúspide. Recomenda-se a realização do exame em repouso, durante todo o esforço, ou logo após o pico da atividade. Contudo, até o momento não existem protocolos universalmente aceitos, os exames são realizados de acordo com a disponibilidade e a experiência de cada serviço.^1 , 34^

A relação E/e’ e a velocidade de pico do jato da insuficiência tricúspide devem ser adquiridas no momento basal e em cada estágio, incluindo o pico do esforço, durante o estágio submáximo e/ou nos primeiros dois minutos da fase de recuperação.^34^

O ecocardiograma sob esforço deve ser considerado anormal quando a relação E/e’ obtida no pico do esforço for ≥15, com ou sem aumento da velocidade de pico da IT para um valor >3,4 m/s. Um aumento isolado na velocidade da IT não deve ser considerado para o diagnóstico de ICFEp, uma vez que essa alteração pode ser meramente causada por uma resposta hiperdinâmica normal ao exercício (aumento do fluxo pulmonar) e na ausência disfunção diastólica do VE. Uma relação E/e’ durante o esforço ≥15 soma 2 pontos ao escore da HFA. Uma relação E/e’ ≥ 15 e a velocidade de pico da IT >3,4 m/s acrescentam 3 pontos ao escore a partir do passo 2 (E). Então, a associação do escore combinado a partir dos passos 2 (E) e 3 (F1) ≥ 5 confirma o diagnóstico de ICFEp.^1 , 34^

Entretanto, algumas limitações são passíveis de ocorrer: a relação E/e’ pode não ser analisável em cerca de 10% dos pacientes durante o esforço submáximo (20W), a velocidade da IT ser mensurável em apenas 50% dos pacientes e cerca de 20% dos pacientes podem ser considerados “falsos positivos”.^31^ Além disso, em nosso país, a disponibilidade de serviços que realizam o ecocardiograma sob esforço físico é bastante escassa, mesmo em cidades com grandes serviços de referência em Cardiologia. No exemplo da Figura 4 , alguns pacientes não se mostram aptos a realizar o procedimento por limitação sintomática ou pela limitação funcional como a coexistência de doenças ortopédicas, articulares, vasculares ou neurológicas.^34^

Finalmente, os dados obtidos a partir da ecocardiografia sob esforço não são suficientes para substituir medidas hemodinâmicas invasivas. Quando o escore persistir <5 pontos ou o ecocardiograma sob esforço não puder ser realizado, recomenda-se a avaliação invasiva quando surgirem dúvidas.^1^ A última diretriz da EACVI^25^ sugere a avaliação hemodinâmica invasiva sob esforço; porém, em nosso país, essa é uma prática utilizada muito raramente e apenas em pacientes específicos. Na prática clínica, pode ser realizada a avaliação invasiva para confirmar a elevação das pressões de enchimento ventriculares esquerdas em repouso (pressão diastólica final do VE ≥16 mmHg) e o diagnóstico de ICFEp.^1^ A avaliação invasiva também deve ser considerada para a exclusão de doença coronária ou em populações específicas.^35^

## Passo 4 (F2): etiologia final

A maioria dos casos de ICFEp está relacionado a fatores de risco e comorbidades comuns. Porém, a possibilidade de uma etiologia subjacente especifica deve sempre ser considerada: cardiomiopatia hipertrófica, miocardite, doenças autoimunes, cardiomiopatias infiltravas, doenças de depósito e endomiocardiofibrose são exemplos.^36 - 38^ Uma vez diagnosticada a ICFEP, a investigação de cada etiologia especifica deve ser pautada pela suspeição clínica e realizada de maneira direcionada na dependência do diagnóstico presuntivo. É fundamental detectar as etiologias específicas, uma vez que esses achados podem se traduzir em terapêuticas específicas. Também é importante considerar que as etiologias não relacionadas ao miocárdio podem apresentar quadro clínico semelhante à ICFEp, como pericardite constritiva, doenças valvares primárias e insuficiência cardíaca de alto débito.^1^

## Limitações, perspectivas e considerações finais

A ICFEp é uma síndrome clínica com múltiplos fatores contribuintes, etiologias e mecanismos fisiopatológicos distintos, de maneira que é impossível a criação de um algoritmo único capaz de realizar o diagnóstico de um grupo tão diversificado de doenças.^39^ Além disso, os resultados dos testes podem ser limitados a este grupo de pacientes, em estágios diferentes da doença e com heterogeneidade de etiologias.^1^

O escore da HFA não atribui pontuação aos fatores de risco clínicos, e sinais e sintomas ao exame físico conforme proposto por autores americanos.^10^ É importante considerar estes fatores, uma vez que, isoladamente, os demais parâmetros dissociados do quadro clínico e exame físico perdem a acurácia diagnóstica. Além disso, outras situações clínicas que não a IC podem levar a elevações dos níveis séricos de biomarcadores como doenças crônicas renais, pulmonares e processos infecciosos, limitando sua aplicação no contexto do paciente com suspeita de IC, uma vez que neste grupo de pacientes a ocorrência destas doenças não é infrequente.^40^

Atualmente, fenótipos distintos tem sido reconhecidos na apresentação clínica dos pacientes com ICFEp, como em relação à caracterização da função atrial esquerda, pressões pulmonares e função ventricular direita.^41^ Nesse contexto, outros parâmetros ecocardiográficos em um futuro próximo poderão ser incorporados ao escore, aumentando a sensibilidade diagnóstica e o detalhamento da fisiopatologia da ICFEp.

Variáveis como os índices de deformação ou ( *strain* ) atrial esquerda apresentam importância crescente na avaliação da função diastólica e nas pressões de enchimento do ventrículo esquerdo. O desenvolvimento de *softwares* dedicados à avaliação do *strain* atrial tem permitido a avaliação mais detalhada da função atrial esquerda e também a análise da rigidez atrial esquerda. Este último é um parâmetro que apresenta correlação logarítmica com as pressões de enchimento do VE e melhor acurácia na predição de valores >15 mmHg da pressão diastólica final do VE em relação à relação E/e’.^42 - 44^ Adicionalmente, outros parâmetros como a deformação do ventrículo direito (global ou da parede livre) desempenham papel promissor no diagnóstico da ICFEp.^45 - 46^

Provavelmente, em um futuro próximo, será possível a análise morfológica não invasiva e relacionada a volumes das cavidades cardíacas, parâmetros hemodinâmicos como volume sistólico, débito cardíaco, pressões de enchimento em associação a novos marcadores de função sistólica e diastólica agregando valor diagnóstico e prognóstico ao significado da FEVE na caracterização da IC.^47 - 49^

O emprego dos métodos modernos para geração de imagem, de maneira integrada, pode fornecer os dados acima mencionados e análises dinâmicas sobre a função arterial, endotelial e perfusão miocárdica, que podem ser acopladas aos dados demográficos, incluindo fatores de risco clássicos e novos biomarcadores com dados sobre proteômica, metabolômica e genética. As informações poderão ser processadas por inteligência artificial, podendo ser úteis na definição de fisiopatologia, diagnóstico, direcionamento terapêutico e predição de desfechos.^47 - 49^

Assim, apesar da elaboração de escores atualizados para o diagnóstico da ICFEp à luz dos novos conhecimentos, principalmente em relação às técnicas ecocardiográficas e aos valores de biomarcadores, ainda são necessários refinamentos e incorporação de mais índices clínicos e ecocardiográficos que permitam não apenas o diagnóstico sindrômico, mas que também orientem a etiologia final dos pacientes com ICFEp.
