# Supplementary material for: Análise Crítica e Limitações do Diagnóstico de Insuficiência Cardíaca com Fração de Ejeção Preservada (ICFEp)
Source: Arq Bras Cardiol. 2022 Jul 7;119(3):470–9. [Article in Portuguese] doi: 10.36660/abc.20210052 (PMC9438546; doi:10.36660/abc.20210052)
Supplement: Translation

# Translation: Critical Analysis and Limitations of the Diagnosis of Heart Failure with Preserved Ejection Fraction (HFpEF)

**Authors:** Viviane Tiemi Hotta, Daniela do Carmo Rassi, José Luiz Barros Pena, Marcelo Luiz Campos Vieira, Ana Clara Tude Rodrigues, Juliano Novaes Cardoso, Felix Jose Alvarez Ramires, Luciano Nastari, Charles Mady, Fábio Fernandes

**Affiliations:** 1 Brazil Instituto do Coração, Hospital das Clínicas da Faculdade de Medicina da Universidade de São Paulo (InCor/HCFMUSP), São Paulo, SP – Brazil; 2 Brazil Fleury Medicina e Saúde, Ecocardiografia, São Paulo, SP – Brazil; 3 Brazil Faculdade de Medicina da Universidade Federal de Goiás, Goiânia, GO – Brazil; 4 Brazil Faculdade de Ciências Médicas de Minas Gerais, Belo Horizonte, MG – Brazil; 5 Brazil Hospital Felício Rocho, Belo Horizonte, MG – Brazil; 6 Brazil Hospital das Clínicas da Faculdade de Medicina da Universidade de São Paulo (HCFMUSP), São Paulo, SP – Brazil; 7 Brazil Hospital Israelita Albert Einstein, São Paulo, SP – Brazil

**Keywords:** Heart Failure/physiopathology, Diagnostic,Imaging, Echocardiography/methods, Natriuretic Peptides

## Abstract

With the increase in the population’s life expectancy and the higher frequency of risk factors such as obesity, hypertension and diabetes, an increase in the prevalence of heart failure with preserved ejection fraction (HFpEF) is expected. However, to date, the diagnosis and treatment of patients with HFpEF remain challenging. The syndromic diagnosis of HFpEF includes several etiologies and diseases with specific treatments but has points in common regarding the clinical presentation, laboratory evaluation related to biomarkers, such as BNP and NT-ProBNP, and echocardiographic evaluation of cardiac remodeling and left ventricular diastolic filling pressures. Extensive randomized clinical trials involving the treatment of this condition have failed to demonstrate benefits to the patient, making it necessary to reflect on the diagnosis, mechanisms of morbidity, mortality and reversibility in this syndrome. In this review, the current concepts, controversies and challenges, especially regarding diagnosis, will be addressed, critically analyzing the *European Heart Failure Association* score for the diagnosis of HFpEF.

## Introduction

It is estimated that in the general population over 60 years of age, approximately 5% of the patients are diagnosed with heart failure with preserved ejection fraction (HFpEF), and the prevalence rate varies between 3.8 and 7.4% among the studies, considering the different methodologies used for the diagnosis.^1^ With the increase in the population’s life expectancy and the higher frequency of risk factors such as obesity, hypertension and diabetes, an increase in the prevalence of HFpEF is expected.^2 - 4^

However, until now, the diagnosis and treatment of patients with HFpEF remain challenging. The syndromic diagnosis of HFpEF includes several etiologies and diseases with specific treatments but has points in common regarding the clinical presentation, laboratory evaluation related to biomarkers, such as BNP and NT-ProBNP, and echocardiographic evaluation of cardiac remodeling and left ventricular diastolic filling pressures.^1^ In contrast to heart failure with reduced ejection fraction (HFrEF), no treatment has yet convincingly shown a reduction in morbidity or mortality in HFpEF, making it necessary to reflect on the diagnosis, mechanisms of morbidity, mortality and reversibility in this syndrome.^5^

In this review, the current concepts, controversies and challenges will be addressed, especially regarding the diagnosis, critically analyzing the *European Heart Failure Association* score for the diagnosis of HFpEF.^1^

### European Heart Failure Association score for the diagnosis of HFpEF

In 2019, the Heart Failure Association (HFA) of the European Society of Cardiology (ESC) published a new statement on the diagnosis of HFpEF, including the role of clinical comorbidities and a system based on a score with updated values of echocardiographic criteria, biomarker measurement, and the role of stress tests ( Table 1 ).^6 - 8^

**Table 1:** Algorithm for the diagnosis of HFpEF, *Heart Failure Association* (HFA) of the *European Society of Cardiology* (ESC)

P	Initial workup	Symptoms and/or signs of HF
	Step 1 (P): Prestest Assessment	Comorbidities / Risk factors
		ECG
		Standard echocardiography
		Natriuretic peptides
		Ergometry / 6 min walking test or cardiopulmonary exercise testing
**E**	Diagnostic workup	Comprehensive echocardiography
	Step 2 (E): echocardiographic and natriuretic peptide score	Natriuretic peptides, if not measured in step 1
**F1**	Advaced workup	Diastolic stress test: exercise stress echocardiography
	Step 3 (F1): functional testing in case of uncertainty	Invasive hemodynamic measurements
**F2**	Etiological workup	Cardiovascularmagnetic resonance
	Step 4 (F2): final etiology	Cardiac or non-cardiac biopsies
		Scintigraphy / CT / PET
		Genetic testing
		Specific laboratory tests

*HF: heart failure; ECG: electrocardiogram; CT: computed tomography; PET: positron emission tomography. Adapted from Pieske B et al.^1^*

The initial evaluation should take into account the anamnesis, while addressing the risk factors and comorbidities and the presence of symptoms and signs on the physical examination of heart failure that suggest the diagnosis of HFpEF, according to the diagram below ( Table 1 ). In this initial phase, blood tests should be performed, including natriuretic peptides (NPs), as well as an electrocardiogram, exercise tests, 6-minute walk tests or cardiopulmonary tests, in addition to echocardiographic evaluations.^1^

The electrocardiogram (ECG) may show signs of left ventricular hypertrophy (Sokolow-Lyon Index ≥ 3.5 mV) and/or left atrial overload, but its main indication is to detect the presence of atrial fibrillation (AF), which is highly predictive of underlying HFpEF.^9 , 10^

The rationale for the use of the score is based on the fact that no noninvasive criterion alone is sufficient for the diagnosis of HFpEF and, therefore, an integrated evaluation of clinical information, measurements of serum levels of natriuretic peptide and evaluation of cardiac structure and function by echocardiography is suggested.^10^ It is important to remember that the cutoff values may vary according to age, gender, body weight, renal function and the presence of atrial fibrillation. Thus, minor and major criteria are recommended according to the degree of change in the presence of the modifying factors described above.^1^

Natriuretic peptide levels in patients with AF rhythm may be up to three times higher than in patients in sinus rhythm; therefore, the cutoff values are different for these two patient populations.^11 , 12^ To date, definitive cutoff values for the diagnosis of HFpEF in patients with sinus rhythm or AF have yet to be established.^1^ The suggested values for the diagnosis of HFpEF are described in Table 2.

### Echocardiographic evaluation

Echocardiography is the cardiac imaging method of choice in the evaluation of patients with signs and symptoms of HF. The echocardiogram allows cardiac functional and anatomical evaluation by measuring the diameters and volumes of the cardiac cavities, estimating the left ventricular mass, and analyzing systolic function by the ejection fraction, in addition to global longitudinal and segmental myocardial function. It is the noninvasive method of choice for the analysis of diastolic function, left ventricular filling pressures and pulmonary artery pressures.^1^

### Morphological criteria

#### Measurements of Left Atrial Volume index (LAVI)

LAVI is related to LV filling pressures and other diastolic function indices, being the most accurate measure of chronic LA remodeling when compared to LA diameter and area.^13 , 14^

In patients without atrial fibrillation (AF) or heart valve disease, LAVI > 34 ml/m^2^is an independent predictor of death, heart failure (HF), atrial fibrillation and ischemic stroke.^15 , 16^ In patients with HFpEF and permanent AF, the LAVI was 35% higher than that of patients with HFpEF in sinus rhythm.^11^ Patients with permanent AF may have higher LAVI even in the absence of diastolic dysfunction. Thus, different LAVI cutoff values are recommended for the diagnosis of HFpEF in patients with sinus rhythm and AF ( Figures 1 and 2 ).^15 , 16^

#### Myocardial thickness measurement and left ventricular mass estimation

In the HFA score, the left ventricular thickness at the end of diastole of the septal and posterior walls are considered morphological criteria for the diagnosis of HFpEF.^1^ These measurements should be obtained preferentially in 2D mode or 2D-guided M mode according to the formula recommended by the American Society of Echocardiography.^17 , 18^

The left ventricular myocardial mass index (LVMI) is defined as the left ventricular mass indexed by the body surface area. Hypertrophy is defined as an increase in LVMI according to the following reference values: ≥ 95 g/m^2^in women and ≥ 115 g/m^2^in men.^17 , 18^ It is also important to consider the calculation of the left ventricular relative wall thickness (RWT).^17 , 18^ The analysis of LVMI and RWT allows the categorization of hypertrophy into concentric (increase in LVMI and RWT >0.42) and eccentric (increase in LVMI and RWT <0.42) or concentric remodeling (normal LVMI and RWT >0.42).^17 , 18^

Remodeling patterns or concentric hypertrophy can be seen in patients with HFpEF. The absence of left ventricular hypertrophy, however, does not exclude the diagnosis of HFpEF.^1^ Thus, for the diagnosis of HFpEF, the criteria described in Figures 1 and 2 are considered.

## Functional criteria

### Measurements of LV Global Longitudinal Systolic Strain (GLS)

The measurement of LV global longitudinal myocardial deformation or strain (GLS) by speckle tracking is independent of the ultrasound insonation angle, conferring an advantage over the strain evaluated by Doppler, being considered the technique of choice.^19^

It is important to consider that equipment by different manufacturers can show variations between GLS values acquired from the same patient. An absolute value of GLS <16% can be considered abnormal and a minor criterion for the diagnosis of HFpEF (see Figure 2 ).^1^ Low values of GLS are predictors of hospitalization for HF, cardiovascular death or cardiorespiratory arrest, showing good correlation with LV stiffness and biomarkers.^19 - 20^

### Measurements on conventional doppler

On conventional Doppler, E-wave measurements are obtained by Pulsed Doppler analysis of the mitral valve to calculate the E/e´ ratio and the tricuspid regurgitation (TR) jet peak velocity is obtained by continuous Doppler. These measurements are important for estimating the increase in filling pressures and, consequently, for the diagnosis of HFpEF.^1 , 19 , 20^

High levels of pulmonary artery systolic pressure (PASP) and right ventricular function reduction are important predictors of mortality in patients with HFpEF. TR jet peak velocity values >2.8 m/s are indirect markers of diastolic dysfunction and are associated with the HFpEF diagnosis.^21 - 24^

### Tissue doppler measurements

The measurements of early diastolic peak velocities (e´ waves) in the septal and lateral walls by pulsed tissue Doppler constitute a key parameter in patients with HFpEF.^1 , 25^ All measurements should represent the mean of three or more consecutive cardiac cycles, and preferably, the measurements of the e´ wave of the septal and lateral velocities should be performed, especially for the calculation of the E/e´ ratio.^25^

The major determinant of the early diastolic velocity of the mitral annulus is LV relaxation. The e´ wave reflects the LV relaxation and is influenced by preload.^26 , 27^ The e´ wave velocity decreases with age; therefore, reference values are recommended according to the age range to calculate the score for the HFpEF diagnosis ( Figures 1 and 2 ).^28^

The average E/e´ ratio of the septal and lateral walls reflects the capillary pressure in the absence of pulmonary stenosis and correlates with left ventricular stiffness and the presence of fibrosis, in addition to being less dependent on age and aging than the e’ wave.^1 , 25 , 29 , 30^ This measure also has diagnostic value during physical effort, being little influenced by volumetric changes but influenced by the left ventricular hypertrophy severity.^1 , 31 - 33^

### Diagnostic evaluation by echocardiographic and natriuretic peptide score

The score includes functional, morphological and biomarker-related domains, with each major criterion assigning 2 points and each minor criterion assigning 1 point to the score (Table 2). It is important to remember that not all parameters of each domain can be analyzed. A total score ≥ 5 points is considered diagnostic for HFpEF, while scores ≤ 1 point indicate a very unlikely diagnosis and make the investigation of differential diagnoses mandatory.^1^ Patients with intermediate scores require an additional complementary assessment (Step 3), as follows. In a structured manner, in practice, steps 1 and 2 can be summarized in the flowchart of Table 2.

Figures 3 and 4 illustrate examples of the score application in real cases.

**Figure 3:** Illustrative example of the diagnostic score application in a patient with suspected HFpEF. A 64-year-old female patient had a history of metabolic syndrome (obesity grade III – Body Mass Index: 35.6, systemic arterial hypertension and diabetes mellitus) and complaints of dyspnea on minimal effort (FC III NYHA). The ECG (above) showed signs of left ventricular hypertrophy according to the Sokolow-Lyon criteria. The TTE displays an interventricular septum and posterior wall thickness of 12 mm and LVMI: 105 g/m2 (1 point). The left atrial volume index estimated at the apical 4C (top left) and apical 2C (top right) views was 48 mL/m2 (2 points). Tissue Doppler shows e’ wave septal velocity = 4 cm/s (bottom left) and lateral e´ wave velocity = 3 cm/s (bottom right) (2 points). Thus, by applying the score for the diagnosis of HFpEF, the patient attained 5 points and, therefore, the HFpEF diagnosis was confirmed.

**Figure 4:** A 78-year-old patient with obesity, systemic arterial hypertension, type 2 diabetes mellitus, paroxysmal atrial fibrillation, in NYHA FC II. On the TTE, LA = 50 mm, LAVI = 38 mL/m2 (2 points), LV mass index: 89 g/m2, LV relative wall thickness = 0.47, non-analyzable PASP and E/e´ ratio = 8.8. BNP = 367 pg/mL (2 points). After applying the score for the diagnosis of HFpEF, the patient attained 4 points and, therefore, had an inconclusive diagnosis of HFpEF.

In the real-life case of Figure 3 , it is important to note that although the patient meets the minor morphological criterion of relative wall thickness >0.42, as she has already received a score within the morphological domain for a major criterion (2 points) due to the dilation of the indexed volume, the minor criterion is not counted within the same domain.

This case is also illustrative because it shows the limitation of the suggested measurements in real cases. In this patient, it was not possible to measure the pulmonary artery systolic pressure due to the absence of tricuspid regurgitation, which is not uncommon in daily practice.

In addition, this patient had limitations in performing the test under exertion due to obesity and degenerative joint abnormalities and did not continue the etiological investigation suggested by the HFA protocol.

It is important to consider that in patients diagnosed with mitral stenosis, the E wave may not reflect diastolic function, as in patients with significant tricuspid regurgitation, in which the tricuspid regurgitation velocity may be reduced due to the equalization between RV and RA, underestimating the measurement of PASP.^25^

## Step 3 (F1): Advanced Evaluation - Functional Test in case of uncertainties

In patients with intermediate diagnostic scores, the performance of complementary evaluation with echocardiography under physical exertion is indicated because many patients only exhibit symptoms on exertion. Thus, symptoms compatible with HFpEF can be confirmed by hemodynamic abnormalities, such as reduced cardiac output, reduced systolic volume and increased LV filling pressures at rest or during physical exertion.^1 , 34^

The stress echocardiography may disclose systolic and diastolic dysfunction during exercise testing. The parameters most frequently used for this analysis when HFpEF is suspected are the E/e´ ratio and the TR jet peak velocity. It is advisable to perform the test at rest and throughout the exertion or immediately after the peak of the exertion. However, to date, there are no universally accepted protocols, and the tests are performed according to the availability and experience of each service.^1 , 34^

The E/e´ ratio and the TR jet peak velocity should be acquired at baseline and at each stage, including the peak of exertion, and during the submaximal stage or during the first two minutes of the recovery phase.^34^

The stress echocardiogram should be considered abnormal if the E/e´ ratio obtained at peak effort is ≥ 15, with or without an increase in the TR peak velocity to a value >3.4 m/s. An isolated increase in the TR peak velocity should not be considered for the diagnosis of HFpEF, as this change may be caused merely by a normal hyperdynamic response to exercise (with increased pulmonary flow) in the absence of LV diastolic dysfunction. An E/e´ ratio during exertion ≥ 15 adds 2 points to the HFA score. An E/e´ ratio ≥ 15 and TR peak velocity >3.4 m/s add 3 points to the score from Step 2 (E). The association of the combined score from Step 2 (E) and Step 3 (F1) ≥ 5 confirms, then, the diagnosis of HFpEF.^1 , 34^

However, some limitations may occur: the E/e´ ratio might not be analyzed in approximately 10% of patients during submaximal effort (20 W), the TR peak velocity was measurable in only 50% of patients, and approximately 20% of the patients could be considered false positive cases.^31^ In addition, in our country, the availability of services that perform echocardiography under physical exertion is very scarce, even in cities with large cardiology referral services. As shown in Figure 4 , some patients are not capable of performing the test under physical exertion, either due to symptomatic limitations or functional limitations, such as the coexistence of orthopedic, joint, vascular or neurological diseases.^34^

Finally, the data obtained from the stress echocardiography are not sufficient to replace invasive hemodynamic measures. When the score remains <5 points or if the stress echocardiogram cannot be performed, the invasive evaluation is recommended in case of doubt.^1^ The last European Association of Cardiovascular Imaging (EACVI) guideline^25^ recommends invasive hemodynamic evaluation under stress; however, this test is very rarely used and only in specific patients in the Brazilian population. In clinical practice, the invasive evaluation can be performed to confirm the elevation of LV filling pressures at rest (LV end-diastolic pressure ≥ 16 mmHg), confirming the HFpEF diagnosis.^1^ An invasive evaluation should also be considered for the exclusion of coronary disease or in specific populations.^35^

## Step 4 (F2): Etiology F final

The majority of HFpEF cases are related to risk factors and comorbidities; however, the possibility of a specific underlying etiology should always be considered, such as hypertrophic cardiomyopathy, myocarditis, autoimmune diseases, infiltrative cardiomyopathies, deposit diseases and endomyocardial fibrosis.^36 - 38^ Once the diagnosis of HFpEF syndrome is made, the investigation of each specific etiology should be guided by clinical suspicion and conducted in a targeted manner, depending on the presumptive diagnosis. The diagnosis of specific etiologies is essential, because these findings can be translated into specific therapies. It is also important to consider that etiologies unrelated to the myocardium may present a clinical picture similar to that of HFpEF, such as constrictive pericarditis, primary valve diseases and high output heart failure.^1^

## Limitations, perspectives and final considerations:

HFpEF is a clinical syndrome with multiple contributing factors, etiologies and distinct pathophysiological mechanisms; hence, it is impossible to create a single algorithm capable of diagnosing such a diverse group of diseases.^39^ In addition, the results of the tests may be limited in this group of patients at different stages of the disease and with heterogeneous etiologies.^1^

The HFA score does not assign a score to the clinical risk factors and signs and symptoms on physical examination as proposed by American authors.^10^ It is important to consider these factors because, individually, the other parameters dissociated from the clinical condition and physical examination lose diagnostic accuracy. Moreover, clinical conditions other than HF, for example, may lead to elevated serum levels of biomarkers, such as chronic kidney and lung diseases and infectious processes, limiting their application in the context of patients with suspected HF, since the occurrence of these diseases in this group of patients is not uncommon.^40^

Currently, distinct phenotypes have also been recognized in the clinical presentation of patients with HFpEF, such as the characterization of left atrial function, pulmonary pressures and right ventricular function.^41^ In this context, other echocardiographic parameters may be incorporated into the score in the near future, increasing the diagnostic sensitivity and detailing the pathophysiology of HFpEF.

Variables such as left atrial strain indices are increasingly important in the evaluation of diastolic function and left ventricular filling pressures. The development of software dedicated to the evaluation of LA strain has allowed a more accurate evaluation of left atrial function and the analysis of left atrial stiffness, a parameter that shows a logarithmic correlation with LV filling pressures and better accuracy in predicting values >15 mmHg of the LV end-diastolic pressure in relation to the E/e´ ratio.^42 - 44^ In addition, other parameters, such as right ventricular deformation (global or free wall), also have a promising role in the diagnosis of HFpEF.^45 - 46^

In the near future, it will probably be possible to perform a noninvasive morphological analysis of cardiac chamber volumes integrated with hemodynamic parameters such as systolic volume, cardiac output and LV filling pressures in association with new markers of systolic and diastolic function, adding diagnostic and prognostic value to the significance of LVEF in the characterization of HF.^47 - 49^

The use of modern imaging methods in an integrated manner can provide the abovementioned data in addition to dynamic analyses on arterial and endothelial function and myocardial perfusion, which can be coupled with the demographic data, including classic risk factors and new biomarkers, with data on proteomics, metabolomics and genetics. This information may be processed by artificial intelligence and may be useful to define the pathophysiology and diagnosis, in addition to therapeutic guidance and outcome prediction.^47 - 49^

Thus, despite the development of updated scores for the diagnosis of HFpEF in the light of new knowledge, especially in relation to echocardiographic techniques and biomarker values, refinements and incorporation of more clinical and echocardiographic indices are still needed, which will allow not only the syndromic diagnosis but also recommendations on the final etiology of patients with HFpEF.
